# Tracheal extubation in deeply anesthetized pediatric patients after tonsillectomy: a comparison of high-concentration sevoflurane alone and low-concentration sevoflurane in combination with dexmedetomidine pre-medication

**DOI:** 10.1186/s12871-017-0317-3

**Published:** 2017-02-21

**Authors:** Meiqin Di, Yuan Han, Zhuqing Yang, Huacheng Liu, Xuefei Ye, Hongyan Lai, Jun Li, Wangning ShangGuan, Qingquan Lian

**Affiliations:** Department of Anesthesiology, Critical Care and Pain Medicine, The Second Affiliated Hospital and Yuying Children’s Hospital of WenZhou Medical University, 109 Xueyuan Western Road, Wenzhou, Zhejiang Province 325027 People’s Republic of China

**Keywords:** Dexmedetomidine, Deep tracheal extubation, Sevoflurane, Pediatric

## Abstract

**Background:**

Dexmedetomidine can facilitate a smooth extubation process and reduce the requirement of sevoflurane and emergence agitation when administrated perioperatively. We aimed to observe the extubation process and the recovery characteristics in pediatric patients undergoing tonsillectomy while anesthetized with either high-concentration sevoflurane alone or low-concentration sevoflurane combined with pre-medication of single dose of intravenous dexmedetomidine.

**Methods:**

Seventy-five patients (ASA I or II, aged 3–7 years) undergoing tonsillectomy were randomized into three equal groups: to receive intravenous saline (Group D0), dexmedetomidine 1 μg/kg (Group D1), or dexmedetomidine 2 μg/kg (Group D2) approximately 10 min before anesthesia. Before the end of surgery, sevoflurane were adjusted to 1.5 times, 1.0 time and 0.8 times the minimal effective concentration in groups D_0_, D_1_ and D_2_, respectively. The sevoflurane concentration for each group was maintained for at least 10 min before the tracheal deep-extubation was performed. The extubation event, recovery characteristics and post-op respiratory complications were recorded.

**Results:**

All tracheal tubes in three groups were removed successfully during deep anesthesia. Nine patients in Group D_0_, three patients in Group D_1_, and two patients in Group D_2_ required oral airway to maintain a patent airway after extubation. The frequency of oral airway usage in groups D_1_ and D_2_ were significantly lower than that in Group D_0_. The percentages of patients with ED and the requirements of fentanyl in groups D_1_ and D_2_ were also significantly lower than those in Group D_0_. The time from extubation to spontaneous eye opening in Group D_2_ was longer than that in groups D_0_ and D_1_. The times of post-anesthesia care unit discharge in groups D_0_ and D_2_ were longer than that in Group D_1_. No other respiratory complications and vomiting were observed.

**Conclusion:**

A single dose of intravenous dexmedetomidine as pre-medication in combination with low-concentration sevoflurane at the end of surgery provided safe and smooth deep extubation condition and it also lowered the emergence agitation in sevoflurane-anaesthetized children undergoing tonsillectomy. Preoperative dexmedetomidine at 1 μg/kg did not prolong postoperative recovery time.

**Trial registration:**

Chinese Clinical Trial Registry (ChiCTR): ChiCTR-IOR-16008423, date of registration: 06 may 2016.

## Background

It is always a challenging task to perform smooth extubation while avoiding coughing, bucking, breath holding, oxygen desaturation, and laryngospasm in children undergoing adenotonsillectomy. Deep tracheal extubation technique has advantages over awake extubation by avoiding some of those complications and it has been safely performed after anesthesia [1]. However, major concerns of airway complications remain hunting in anesthesiologist’s mind, particularly in young children undergoing airway surgeries, and with co-existing obstructive airway diseases and when anesthetized with inhaled volatile agent, such as sevoflurane.

Dexmedetomidine, a highly selective α-2 adrenergic agonist, is widely employed perioperatively in children [2, 3] and it can reduce the requirement for anesthetics [4, 5], as shown by a previous study that dexmedetomidine could produce a dose-dependent decrease of sevoflurane in children, from 33 to 60% [6]. Guler et al. have found that a single-dose injection of dexmedetomidine facilitated smooth awake extubation by attenuating the extubation-induced airway-circulatory reflexes in children [7, 8].

In this study, we hypothesized that dexmedetomidine, combined with a low-concentration sevoflurane would create a smooth deep-extubation condition compared to the high-concentration sevoflurane anesthesia without dexmedetomidine in children undergoing adenotonsillectomy. The emergence characteristics, recovery time and incidence of airway complications were all of interest to be observed.

## Methods

The study was approved by the Hospital Ethics Committee of the Second Affiliated Hospital and Yuying Children's Hospital of WenZhou Medical University. After a written informed consent was obtained from the parents, a total of 75 children, aged 3–7 years old, American Society of Anesthesiologists (ASA) physical status I or II, scheduled to undergo an adenotonsillectomy during the period of May 2016 to July 2016, were enrolled in this observational study (trial registry identifier, ChiCTR-IOR-16008423). Patients with suspected difficult airway, current upper respiratory infections, asthma, mental diseases, or other congenital and neurological diseases were excluded from the study.

All patients were required to follow the ASA fasting guideline [9]. In the morning on the day of surgery, an intravenous (IV) line was placed in the ward, and patients were sent to a pre-anesthesia holding room approximately 20 min before surgery in the presence of one parent. Prior to any pre-medication, noninvasive blood pressure, ECG, oxygen saturation (SPO_2_) and heart rate were measured as baseline and then, were recorded continuously. Subjects were randomly allocated to one of three groups (Group D_1_, Group D_2_ and Group D_0_, *n* = 25 per group) by a computer-generated table of random numbers. Prior to anesthesia induction, patients in Group D_1_ and Group D_2_ received IV infusion of dexmedetomidine (4 μg/mL normal saline) at 1 μg/kg and 2 μg/kg over 10 min respectively. Patients in Group D_0_ received saline infusion over 10 min.

Upon the completion of premed infusion, the patients were transferred to the operating room. The monitoring of vital signs were initiated and then, continued after. Anesthesia was induced with sevoflurane (8%) in oxygen at 5 L/min. When the pupils were deemed to be small and central, the trachea intubation was performed without muscle relaxants. Respiratory rate, tidal volume, end-tidal carbon dioxide partial pressure (ETCO_2_), and minimum alveolar concentration (MAC) of sevoflurane were monitored. Patients who received rocuronium (0.6 mg/kg) for failed intubation during the first 30 s attempt, or who were persistently coughing after tracheal intubation were excluded from the study. Prior to the surgical incision, fentanyl (0.5 μg/kg), ondansetron hydrochloride (0.1 mg/kg) and dexamethasone (0.2 mg/kg) were administrated to all children, and 0.2% ropivacaine (0.25 mg/kg) with 1:200 000 epinephrine was injected locally to the tonsil bed to provide additional postoperative analgesia when surgery was finished. An adequate depth of anesthesia was maintained with sevoflurane, and the patients were allowed breathing spontaneously throughout. Before the end of surgery, sevoflurane were set to 1.5, 1.0 and 0.8 MAC in groups D_0_, D_1_ and D_2_, respectively, and were maintained at the same level for at least 10 min to achieve equilibration between the alveolar and brain. The concentration of sevoflurane for group D_0_ was chosen based on the precious study in which sevoflurane at 1.5 MAC may provide a satisfactory deep extubation condition in children [10]. The concentrations of sevoflurane for group D_1_ and group D_2_ were determined based on the study which was conducted in children, 1.0 μg/kg dexmedetomidine followed by a continuous infusion of 0.5 μg/kg/h reduced ED50TI (50% excellent tracheal intubation conditions) of sevoflurane by 33%, and 2.0 μg/kg dexmedetomidine followed by a continuous infusion of 1.0 μg/kg/h reduced ED50TI of sevoflurane by 60% [6], and based on the results of our pilot study.

Adequate spontaneous respiration was defined as a normal ETCO_2_ waveform and an ETCO_2_ concentration less than 6.0 kPa [11]. The ventilation was assisted manually when ETCO_2_ concentration was over 7.2 kPa.

After surgery, patient was positioned on his or her lateral side, the oropharynx was gently suctioned, and the tracheal tube cuff was deflated. Once a stable spontaneous respiratory pattern was confirmed by ETCO_2_ monitoring, the endotracheal tube was removed gently and quickly. Sevoflurane was discontinued and oxygen (8 L/min) was administered via a facemask immediately after extubation. An oral airway was placed only if the patient had signs of obstructed airway. In addition, propofol 2 mg/kg and continuous positive airway pressure (CPAP) would be used if patients developed breath holding or laryngospasm. Smooth tracheal extubation was defined as no gross purposeful muscular movement, such as coughing, breath holding or laryngospasm within 1 min immediately after tracheal tube removal [12]. Quality of extubation was assessed by using a 5 point rating scale [13] (Extubation Quality Score): 1 = no coughing; 2 = minimal coughing (1 or 2 times); 3 = moderate coughing (3 or 4 times); 4 = severe coughing (5–10 times) and straining; and 5 = poor extubation, very uncomfortable (laryngospasm and coughing > 10 times). A research observer who was blinded to the groups and the drugs which the patient received was assigned to evaluate the quality of extubation and the respiratory complications (breathe holding, laryngospasm, bronchospasm and hypoxemia) and comply the data. Respiratory and hemodynamic profiles were continuously monitored throughout the procedure and until 5 min after extubation. Anesthesia time (time from sevoflurane induction to sevoflurane discontinuation) and surgery time were registered. The requirement of oral airway and airway support were both noted.

Patients were transferred to the post-anesthesia care unit (PACU) positioning on their lateral side when a patent airway and adequate spontaneous ventilation were assured after extubation. The adequacy of the airway was assessed by the criteria of SpO_2_ > 97% with 100% oxygen, clear breath sounds and normal chest wall movement. The pediatric anesthesia emergence delirium scale (PAED) was used to diagnose the emergence agitation (EA) in the PACU [14]. EA was defined to a total score > 10. AS a rescue analgesic, fentanyl (0.5 μg/kg) was administrated to the children who had EA. Patients were discharged from the PACU when they had an Aldrete score of 9–10, present of being calm, no pain and nausea [15]. PAED score and the incidence of emergence agitation, postoperative vomiting, any respiratory complications and the fentainyl administration were recorded. The recovery time (from extubation to spontaneous eye opening) and the actual time to discharge from PACU were both noted.

Fan reported that the incidence of smooth extubation was 88% in adult patients receiving 1.0 MAC sevoflurane combined with dexmedetomidine 0.7 μg/kg [12]. A power analysis performed before the initiation of our study suggested that a sample size of 24 patients for each group should be adequate to detect a 70% smooth extubation at the 0.05 level with a power of 0.8.

The results were expressed in terms of [mean ± standard deviation (SD), n (%)] unless otherwise noted. Parametric data among groups were analyzed using One-way analysis of variance and Mann–Whitney rank-sum test, depending on the distribution of the data. Nominal data were analyzed using either *χ* ^2^ or Fisher’s exact tests. *P* < 0.05 was considered statistically significant.

## Results

**Fig. 1:**
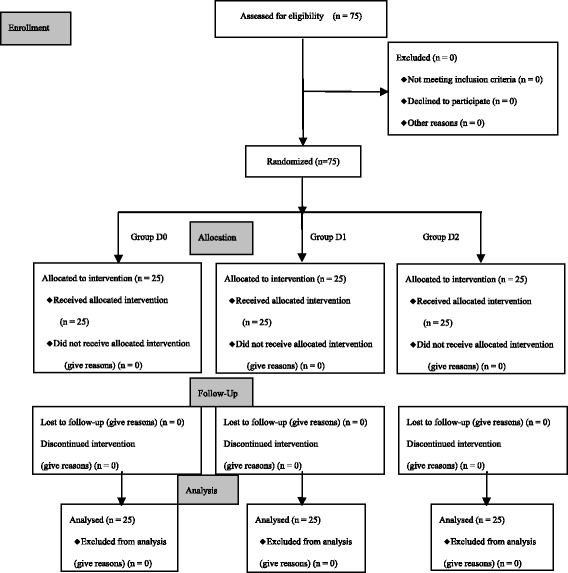
CONSORT flow diagram

A total of 75 eligible children were grouped into this study (see Fig. 1, CONSORT flow diagram). There were no dropouts or protocol violations and complete datasets were available for all children. The demographic data (age, sex and weight), the durations of surgery and anesthesia were similar in three groups, as presented (see Table 1).

**Table 1:** Demographics and clinical characteristics

Variable	Group D_0_	Group D_1_	Group D_2_
	(*n* = 25)	(*n* = 25)	(*n* = 25)
Age (y)	5.3 ± 1.2	4.7 ± 1.0	4.9 ± 1.2
Sex (male/female)	14/11	10/15	13/12
Weight (kg)	19.8 ± 2.9	18.6 ± 2.8	19.4 ± 4.2
Anesthesia duration (min)	27.0 ± 4.2	27.0 ± 3.7	28.0 ± 2.7
Duration of surgery (min)	18.1 ± 3.5	18.8 ± 3.1	18.0 ± 2.9

Data are expressed as mean ± standard deviation (SD) (One-way ANOVA test) or number of patients (Chi Square test)

Group D_0_ saline group, Group D_1_ dexmedetomidine group (1 μg · kg^−1^), Group D_2_ dexmedetomidine group (2 μg · kg^−1^)

All tracheal tubes in the three groups were removed successfully during deep anesthesia. Only two patients developed minimal cough in group D_0_ and none in Group D_1_ and Group D_2._ After extubation, nine patients in Group D_0_, three in Group D_1_, and two in Group D_2_ required an oral airway. The frequency of oral airway insertion in groups D_1_ and D_2_ were significantly lower than that in Group D_0_, *P < 0.05*. No breath holding, laryngospasm, bronchospasm and hypoxemia were observed during extubation and after the extubation in any of the groups. No re-intubation was required, as shown (see Table 2). The hemodynamics profiles and respiratory pattern were stable during tracheal extubation in all groups. There were no significant differences in respiratory rate, tidal volume, ETCO_2_, MAP, heart rate and SPO_2_ before anesthesia induction, just before extubation, during extubation, and at 1 and 5 min after extubation among three groups.

**Table 2:** Extubation characteristics, use of oral airway

Study group	Group D_0_	Group D_1_	Group D_2_
	(*n* = 25)	(*n* = 25)	(*n* = 25)
Smooth extubation	25(100.0)	25(100.0)	25(100.0)
Extubation Quality Score			
no coughing	23(92.0)	25(100.0)	25(100.0)
minimal coughing	2(8.0)	0	0
moderate coughing	0	0	0
severe coughing	0	0	0
poor extubation	0	0	0
Use of oral airway	9(36.0)	3(12.0)*	2(8.0)*

Data are expressed as number of patients (%) (Chi-Square test)

Group D_0_ saline group, Group D_1_ dexmedetomidine group (1 μg · kg^−1^), Group D_2_ dexmedetomidine group (2 μg · kg^−1^)

*P < 0.05 vs. Group D_0_

In the PACU, 11 patents in Group D_0_ exhibited ED and were treated with fentanyl. No patient in Group D_1_ and Group D_2_ had EA and required fentanyl administration. The percentages of patients with ED and the requirements of fentanyl in groups D_1_ and D_2_ and were both significantly lower than those in Group D_0_. The recovery time in Groups D_2_ and D_0_ were longer than that in group D_1_. Furthermore, the recovery time in Group D_2_ was also longer than that in group D_0_. The times of PACU discharge were comparable in Group D_0_ and Group D_2_, both longer than that in Group D_1_, as shown (see Table 3). No respiratory complications and the incidence of vomiting were observed in PACU.

**Table 3:** Recovery variables in the post-anesthesia care unit

Study group	Group D_0_	Group D_1_	Group D_2_
	(*n* = 25)	(*n* = 25)	_0−_(*n* = 25)
Emergence agitation (cases)	6(24)	0*	0*
Requirements of fentanyl (μg)	4.5 ± 0.9	0*	0*
Time from extubation to spontaneous eye opening (min)	23.6 ± 5.0^#^	19.8 ± 4.3	27.5 ± 5.0*^#^
Time to discharge from PACU (min)	33.7 ± 7.4^#^	25.5 ± 5.0	32.8 ± 4.9^#^

Data are expressed as mean ± standard deviation (SD) (One-way ANOVA test) or number of patients (%) (Chi Square test)

Group D_0_ saline group, Group D_1_ dexmedetomidine group (1 μg · kg^−1^), Group D_2_ dexmedetomidine group (2 μg · kg^−1^)

*P < 0.05 vs. Group D_0_, ^#^ *P < 0.05* vs. Group D_1_

## Discussion

Our study results showed that a single intravenous injection of dexmedetomidine (1 μg/kg or 2 μg/kg) as pre-medication allowed safe and smooth tracheal extubation in children who were undergoing tonsillectomy and anesthetized with low concentrations of sevoflurane, and it also effectively prevented emergence agitation from anesthesia. And dexmedetomidine at 1 μg/kg in combination with sevoflurane 1.0 MAC did not delay discharge time from anesthesia.

Airway surgery in children, particularly tonsillectomy posts a higher airway complications perioperatively which frequently occurs following tracheal extubation [16]. There has been ongoing debate regarding the tracheal extubation strategies. Awake extubation technique may increase the risk of complications, such as tonsillar hemorrhage, over deep extubation, but, several other studies did not find any differences of the perioperative respiratory complications between two approaches [17, 18]. However, patients who experience the deep extubation will have increased overall comfort as opposed to strenuous coughing and gagging over the tube, less risk of hemodynamic swings, less concern of tonsil bleeding and less cough after extubation [17]. In addition, deep extubation could improve recovery, and prevent wound dehiscence and bronchial spasm [10]. While an extremely unpleasant experience of awake extubation may increase the incidence of postoperative emergence agitation [19], which has untoward impact on children in their early recovery [20].

Dexmedetomidine is a selective α_2_-adrenoceptor agonist. Intravenous dexmedetomidine completely blocked histamine-induced bronchoconstriction in dogs [21]. Dexmedetomidine might benefical to decrease airway reacitivity. Fan et al. found that, after intravenous injection of dexmedetomidine at 0.7 μg/kg 10 min before the end of surgery, adjunctive sevoflurane could ensure deep extubation without complications during spontaneous breathing in adults [11]. Some clinicians have used dexmedetomidine in combination with propofol to achieve the adequate anesthesia plane for deep extubation in children after airway reconstruction [22]. The purpose of our study was to find out whether dexmedetomidine in combination with low sevoflurane was able to facilitate deep extubation in children undergoing tonsillectomy.

For inhalation anesthesia, a certain concentration of inhaled anesthetic agent is required to meet the criteria of deep extubation. Studies have shown that in the absence of analgesic drugs, sevoflurane at 1.5 MAC may provide a satisfactory deep extubation condition in children [10]. The dose of sevoflurane required could be significantly lowered if dexmedetomidine was given as a pre-medication [3, 4]. He et al. found that a single injection of dexmedetomidine at 1 μg/kg before anesthesia induction, then followed by continuous infusion of dexmedetomidine at 0.5 μg/kg/h could reduce ED_50_TI of sevoflurane by 33%, and a single injection of dexmedetomidine at 2 μg/kg before anesthesia induction followed by continuous infusion of dexmedetomidine at 1 μg/kg^/^h reduced ED_50_TI of sevoflurane by 66%, showing that dexmedetomidine played a role in a dose-dependent manner [6]. The distribution half-life of dexmedetomidine was 3.2-5.5 min, and the elimination half-life of dexmedetomidine is 2 h in children. Evidence showed that dexmedetomidine at 0.4 μg/kg before anesthesia induction could assist a minor ophthalmological clinic surgery [3]. An uncomplicated tonsillectomy is a common short-time surgery in children and may be completed within 30 min. In this study, intravenous saline, dexmedetomidine 1 μg/kg and dexmedetomidine 2 μg/kg were given prior to anesthesia induction in group D_0_, D_1_ and D_2_ respectively. A smooth extubation was observed in all of the three groups even a lower sevoflurane was used in group D1 (MAC 1.0) and group D_2_ (MAC 0.8) compared to the sevoflurane-only D_0_ group (MAC 1.5). Moderate to severe cough was not observed, too. Our results indicated that Dexmedetomidine can facilitate deep extubation process with less inhaled agent required.

Emergence agitation after general anesthesia with sevoflurane is another major concern in children undergoing ENT surgery, and the causes are multi-factor directed. Among those, a brief exposure of sevoflurane anesthesia is one of the major contributors. The incidence of emergence agitation is high (up to 80%) depending on the definitions and methods of evaluation [23, 24]. Deep extubation technique has been conducted by some investigators, aiming to reduce emergence agitation after sevoflurane anesthesia. However, the results from a few other studies showed that the incidence of emergence agitation in deep extubation was similar to that after awake extubation [19]. It has been known that preoperative anxiety was closely related to the increased incidence of postoperative emergence agitation [25]. Theoretically, anti-anxiety pre-medication would help to reduce the incidence of post-operative agitation and delirium. DEX is widely used in children as pre-medication to alleviate preoperative anxiety and nervousness, to optimize anesthesia induction by sevoflurane, and to improve postoperative recovery after anesthesia. Yao et al. [4] confirmed that intranasal dexmedetomidine at 2 μg/kg relieved the preoperative anxiety and decreased postoperative agitation. Bhadla et al. reported that a bolus injection of dexmedetomidine at 0.4 μg/kg before surgery could significantly lower the incidence of emergence agitation after ophthalmological minor clinic surgery [3]. Our results showed that patients pre-medicated with a single intravenous dexmedetomidine at 1 μg/kg in Group D_1_ and dexmedetomidine at 2 μg/kg in Group D_2_ had significantly lower incidence of postoperative emergence agitation compared to patients in group D_0_. Thus, the data suggest that preoperative dexmedetomidine administration do reduce postoperative emergence agitation in a wide dosing range.

The sedation effect of dexmedetomidine was dose dependent. The higher the dose of dexmedetomidine goes, the better the sedation will be, but the concern is that a high dose of dexmedetomidine may prolong the postoperative recovery time. In addition, sevoflurane concentration is also related to recovery time after anesthesia. In the present study, the median recovery time was 23.6 min in Group D_0_, which was comparable to the reported by Valley et al. [10]. The time in Group D_1_ was 19.8 min, which was a rapid awakening, whereas the time in Group D_2_ was much longer (27.5 min) than in Group D0 and Group D1. The difference might be due to the different concentrations of sevoflurane and different doses of dexmedetomidine pre-medication between the groups. The discharge time in patients from Group D_0_, and D_2_ was comparable, but much longer than in Group D_1_ patients. The presumptive explanation would be that patients in group D_0_ received additional fentanyl for treatment of emergence agitation and patients in group D_2_ had higher dose of dexmedetomidine (2 μg/kg).

The potential complications during deep extubation include aspiration, airway obstruction, oxygen desaturation, and airway spasm. In this study, the absence of airway responsiveness and the continuation of regular and spontaneous respiration after laryngopharyngeal suction and the deflation of the endotracheal tube cuff were used to determine whether patients were ready for deep extubation. The recorded respiratory frequency, tidal volume, and ETCO_2_ prior to extubation were comparable among groups, which was consistent with the perception of dexmedetomidine having minimal effect on respiration [26]. Dexmedetomidine has a very safe therapeutic window with respect to respiratory depression, even if dexmedetomidine at a high dose [26]. However, 36% of children in Group D_0_ required an oral airway after extubation, and this rate was significantly higher than patients in groups D_1_ and D_2_, which may be related to muscle relaxation induced by a high concentration of sevoflurane [27].

Rapid bolus i.v. administration of dexmedetomidine has possible hemodynamic side effect, it may alter the hemodynamics, for example, by slowing heart rate and modifying blood pressure, which are potential disadvantages of dexmedetomidine. However, these adverse effects can be minimized by slow IV infusion of dexmedetomidine over 10 min. Our results showed that the hemodynamics remained stable during extubation in the three groups.

One of the limitations for this study was that the plasma concentration of dexmedetomidine was not monitored during anesthesia. The correlation between the length and depth of sedation and the hemodynamic changes of dexmedetomidine in blood could not be analyzed.

## Conclusions

In children undergoing tonsillectomy, single intravenous dexmedetomidine pre-medication at 1 μg/kg or 2 μg/kg could facilitate deep extubation in the presence of inhaled low-concentration sevoflurane, which also reduces the incidence of emergence agitation. Dexmedetomidine at 1 μg/kg pre-medication did not prolong postoperative recovery time.

## Abbreviations

ASA: American society of anesthesiologists
CPAP: Continuous positive airway pressure
EA: emergence agitation
ED_50_TI: 50% excellent tracheal intubation conditions
ETCO2: End-tidal carbon dioxide
MAC: Minimum alveolar concentration
PACU: Post-anesthesia care unit
PAED: Pediatric anesthesia emergence delirium scale
SPO_2_: oxygen saturation.
